# Evaluation of the Pro-, Anti-Inflammatory, and Anabolic Effects of Autologous Platelet-Rich Gel Supernatants in an *in vitro* Coculture System of Canine Osteoarthritis

**DOI:** 10.1155/2022/3377680

**Published:** 2022-04-11

**Authors:** Miller Gallego, Catalina López, Jorge U. Carmona

**Affiliations:** ^1^Grupo de Investigación Terapia Regenerativa, Departamento de Salud Animal, Universidad de Caldas, Manizales, Colombia; ^2^Grupo de Investigación Patología Clínica Veterinaria, Departamento de Salud Animal, Universidad de Caldas, Manizales, Colombia

## Abstract

There are scarce *in vitro* studies indicating the basic mechanisms of why platelet-rich plasma (PRP) is useful in the clinical management of dogs with naturally occurring OA. *Methods*. Cartilage and synovial membrane explants from six dogs were challenged with lipopolysaccharide (LPS) and cultured for 48 h with platelet-poor gel supernatant (PPGS) and platelet-rich gel supernatant (PRGS) at concentrations of 25 and 50%, respectively. The tissue explants challenged with LPS were cocultured over 48 h and culture media were sampled at 1 and 48 h for the determination of IL-1*β*, IL-10, hyaluronan, TGF-*β*1, and PDGF-BB by ELISA. *Results*. IL-1*β* concentrations were significantly higher in tissue explant groups cultured for 48 h with PRGS at 50% and with PPGS at 25% when compared to the remaining experimental groups at any time. IL-10 and HA presented similar concentrations in all evaluated groups at any time. TGF-*β*1 and PDGF-BB presented higher concentrations in the culture media of tissue explants cultured with PPGS and PRGS at 50%, which diminished with time. *Conclusions*. Both PPGS and PRGS at both concentrations showed a limited biological effect on cartilage and synovial membrane explants in coculture with LPS. Even PPGS at 25% and PRGS at 50% exhibited proinflammatory effects on these tissues at 48 h.

## 1. Introduction

Osteoarthritis (OA) is the most frequent pan-articular disease observed in human beings and animals such as dogs [1]. This inflammatory/degenerative condition is characterized by progressive articular cartilage damage, subchondral bone remodeling, synovitis, and intra- and peri-articular soft tissue deterioration, which is sometimes accompanied by ligament rupture that induces additional mechanical damage secondary to joint instability [2, 3].

OA can appear as a primary (spontaneous) disease or be associated with both ligament lassitude or rupture, thereby producing joint instability and joint incongruence [4]. As in humans, OA in dogs is increasing dramatically due to the increased life expectancy of pets worldwide. Studies conducted in the UK and USA have found that OA could affect between 6.6 and 20% of dogs over 1 year of age [1, 5, 6], which could represent a population of 15 million dogs in the USA alone [3].

The main clinical signs related to OA include joint effusion and lameness, which are indicative of pain. This condition produces the loss of joint function and reduces patients' quality of life. Thus, the therapeutic goal for the management of this disease is centered on controlling pain and avoiding progressive joint deterioration [3, 4].

Regardless of the advances in knowledge of OA pathophysiology [2], this disease continues to be managed with diverse symptomatic treatments, such as the systemic or intra-articular injection of analgesics and anti-inflammatories, oral nutraceuticals accompanied by physical therapy, and lifestyle changes that could include exercise restriction and weight loss [3, 7]. However, while this disease could progress in some cases, with surgical joint replacement being a potential final option, euthanasia also appears as the most drastic decision for many owners due to budget restrictions or concerns related to their dog's quality of life [3].

Regenerative medicine is emerging as a new therapeutic approach aimed—among the better cases—to restore the structure and function of organs or tissue damaged by trauma, inflammation, or a degenerative process. There are several encouraging regenerative therapies for the clinical management of OA in dogs, such as those using stem cells [8–12] and platelet-rich plasma (PRP) [13–17]. However, from economic and technical perspectives, PRP could be a more universal and affordable orthobiologic than stem cell therapy for OA management—especially in third world countries with limited access to stem cell technology.

To the best of the authors' knowledge, there is a scarcity of published studies indicating the basic mechanisms behind why PRP is useful in the clinical management of dogs with naturally occurring OA. In line with this, we present a study that evaluated the anti-inflammatory and anabolic effects of two autologous hemocomponents (platelet-rich gel supernatant (PRGS) and platelet-poor gel supernatant (PPGS)) in an *in vitro* system of canine osteoarthritis, which included the coculture of canine cartilage and synovial membrane explants challenged with lipopolysaccharide (LPS). Thus, we measured and compared the released concentrations of the anti-inflammatory and anabolic growth factors transforming growth factor beta 1 (TGF-*β*_1_) and platelet-derived growth factor-BB (PDGF-BB, a proinflammatory cytokine, and interleukin 1 (IL-1)), as well as the anti-inflammatory mediator IL-10 and the anabolic indicator hyaluronan (HA), in the culture media of an OA system cultured with autologous PRGS and PPGS concentrations (25 and 50%) over 48 h. We hypothesized that PRGS at any concentration would diminish the release of IL-1 and increase the release of IL-10 and HA.

## 2. Materials and Methods

This study was approved by the Committee for Animal Experimentation of the authors' institution. The study was conducted according to international welfare guidelines for animal experimentation and the panel on euthanasia of the AVMA [18], while also considering the rule in the Colombian act for dangerous dogs [19].

### 2.1. Animals

The biological material (blood, cartilage, and synovial membrane) used in this study was obtained from six clinically healthy dogs (four females and two males) between 1.5 and 3.5 years old, with a mean weight of 24.2 ± 2.87 kg. According to Colombian law, all included animals were considered dangerous dogs that were abandoned by their owners or seized. The animals were sedated with xylazine and butorphanol and then received an intravenous overdose of sodium pentobarbital [18].

### 2.2. Blood Collection and Hemocomponent Processing

Whole blood was collected from each sedated dog before euthanasia. Briefly, the blood was aseptically obtained via jugular venipuncture and deposited in a 450 mL transfusion bag containing CPDA-1 as an anticoagulant. A 10 mL blood sample was used to perform automated blood cell counts.

#### 2.2.1. Platelet-Rich Plasma and Plasma Procurement

First, 10 mL blood samples were aseptically deposited in 15 mL sterile Falcon tubes. Then, five 10 mL Falcon tubes were used for PRP processing and the five additional tubes were used for PPP procurement. The leftover blood was used for other experiments unrelated to this research or clinical purposes in the Veterinary Medical Teaching Hospital of the authors.

PRP was obtained by a simple spinning of the Falcon tubes at 191 g for 6 min. After centrifuging each blood tube, there was a differentiation between the packed cell volume (∼40%) and plasma (∼60%). A sterile pipette was then introduced near the buffy coat and 50% of the plasma was gently aspirated to avoid the over-aspiration (contamination) of red blood cells (RBCs) and white blood cells (WBCs)—especially polymorphonuclear cells. The resulting plasma fraction (∼15 mL) was considered PRP, which was then activated with calcium gluconate over 3 h at 37°C. Thereafter, the PRGS was stored at −80°C for later use in the in vitro explant tissue coculture and for the basal determination of IL-1*β*, IL-10, HA, TGF-*β*_1_, and PDGF-BB.

PPP was obtained by centrifuging an additional five Falcon tubes at 3500 g for 10 min. The 50% top plasma fraction was used for the experiment. This fraction was activated with calcium gluconate over 3 h at 37°C to obtain platelet-poor gel supernatant (PPGS), which was managed similarly to PRGS.

### 2.3. Cartilage and Synovial Membrane Harvesting

After euthanasia, the entire femoral-tibial region from both legs of each dog was aseptically prepared for joint tissue harvest. After surgical stifle exposition, several cartilage slices free from their calcified region were obtained with a scalpel from lateral and medial condyles. Furthermore, the surrounding condyle joint capsule was dissected for obtaining synovial membrane explants (SMEs). Cartilage explants (CEs) and SMEs were obtained by using a 4 mm circular biopsy punch. Notably, SMEs were gently released from the circular joint capsule explants previously obtained with the biopsy punch.

### 2.4. *In vitro* Tissue Explant Coculture and Study Design

Both the CEs (*n* = 12) and SMEs (*n* = 12) from each dog were rinsed with phosphate-buffered saline solution and stabilized in Dulbecco's modified eagle medium (DMEM) (high glucose, 4500 mg/L) with L-glutamine and sodium bicarbonate free from sodium pyruvate (DMEM, Lonza Group Ltd, Basel, Switzerland) and supplemented with streptomycin (100 *μ*g/mL) and penicillin (100 *μ*g/mL) without the addition of serum. Cultures were incubated in a 5% CO_2_ and water-saturated atmosphere for 24 h and then replaced with fresh culture media. At this time point, a part of the CEs and SMEs was challenged with 100 ng/mL of LPS (Sigma-Aldrich, St Louis, MO, USA) to induce the inflammatory/catabolic damage of these tissues, as described in other studies [20, 21].

**Figure 1:**
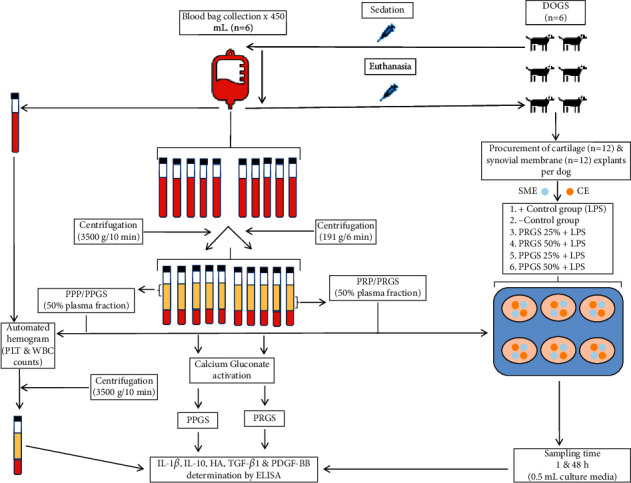
Study design and methodology. HA, hyaluronan; IL, interleukin; LPS, lipopolysaccharide; PLT, platelet; PDGF-BB, platelet-derived growth factor isoform BB; PPP/PPGS, platelet-poor plasma/platelet-poor gel supernatant; PRP/PRGS, platelet-rich plasma/platelet-rich gel supernatant; TGF-*β*_1_, transforming growth factor beta 1; WBC, white blood cell.

Six experimental groups (with two CEs and two SMEs each in coculture) were included. Cocultured tissue explants were performed in six-well plates (Corning, Costar, TC-Treated Multiple Well Plates, Merck KGaA, Darmstadt, Germany) with a total volume of 3 mL per well, considering the final concentration of the PRGS and PPGS assayed in the culture media. The study design included the evaluation of two cocultured tissue explant control groups (one with the addition of LPS and one without LPS) without the addition of any hemoderivatives, as well as four cocultured tissue explant LPS-challenged groups cultured with PRGS and PPGS at two concentrations (25 and 50%). All tissue explant groups were cocultured over 48 h. Samples of culture media (0.5 mL) were obtained at 1 h and 48 h. These samples were obtained, aliquoted, and frozen at −80°C for subsequent determination of the biomolecules.  Figure 1 shows the study design and methodology.

### 2.5. Cytokine, Hyaluronan, and Growth Factor Measurement

IL-1*β*, IL-10, HA, TGF-*β*_1_, and PDGF-BB concentrations were measured in PRGS, PPGS, and the culture media (at 1 and 48 h) of the tissue explant groups in coculture. These mediators were measured by ELISA in duplicate. All proteins were assayed using commercial ELISA development kits from R&D Systems (Minneapolis, MN, USA). IL-1*β* (Canine IL-1 Beta/IL-1F2 DuoSet, DY3747) and IL-10 (Canine IL-10 DuoSet, DY735) were assayed with species-specific canine antibodies for these mediators and HA (Hyaluronan DuoSet, DY3614) was determined using a multispecies detection ELISA kit. TGF-*β*_1_ (Human TGF-*β*1 DuoSet, DY240E) and PDGF-BB (Human PDGF-BB DuoSet, DY220) were determined using human antibodies because there is a high sequence homology between these proteins in mammals such as humans and canines [22, 23]. Furthermore, similar ELISA antibodies have been used for the same purposes in other canine PRP studies [24, 25]. The standards provided for each ELISA kit were used to plot each standard curve according to the manufacturers' instructions. Absorbance readings were performed at 450 nm.

### 2.6. Statistical Analysis

The Shapiro–Wilk test was used to assess the fit of the data set to a normal distribution (goodness of fit). All evaluated parameters demonstrated a normal distribution (*P* > 0.05), except WBC counts in both hemocomponents. PLT in whole blood, PRP, and PPP were compared using a one-way analysis of variance (ANOVA) followed by a post hoc Tukey test. WBC counts were analyzed using a Kruskal–Wallis test followed by a Mann–Whitney *U* test. The GF, cytokines, and HA contained in PPGS and PRGS were compared using a *t*-test for unpaired samples.

A generalized linear model (GLM) univariate analysis was followed (when necessary) by a Games–Howell test, which was used to compare biomolecule concentrations in culture media from each experimental group at 1 and 48 h. The statistical model evaluated the interactions over time at two levels (1 h and 48 h) and experimental group at six levels (control group, control group plus LPS, 25% and 50% PPG, and 25% and 50% PRGS). *P* < 0.05 was accepted as statistically significant for all tests.

## 3. Results

### 3.1. Cell Counts in Whole Blood and Hemoderivatives

**Figure 2:**
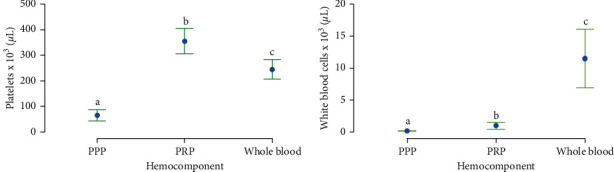
Cell concentrations in whole blood and hemoderivatives. (a) Mean (±SD) platelet concentrations in whole blood, PRP, and PPP. (b) Mean (±SD) leukocyte concentrations in whole blood, PRP, and PPP. Different lower-case letters represent significant (*P* < 0.05) differences between hemocomponents for platelet and leukocyte concentration. HA, hyaluronan; IL, interleukin; LPS, lipopolysaccharide; PLT, platelet; PDGF-BB, platelet-derived growth factor isoform BB; PPP/PPGS, platelet-poor plasma/platelet-poor gel supernatant; PRP/PRGS, platelet-rich plasma/platelet-rich gel supernatant; TGF-*β*_1_, transforming growth factor beta 1; WBC, white blood cell.

Platelet counts were significantly different in whole blood, PRP, and PPP, with the highest counts for PRP, followed by whole blood and PPP (Figure 2(a)). A similar significant finding was observed for WBC counts in whole blood and both hemoderivatives. However, whole blood showed the highest WBC concentrations, followed by PRP and PPP (Figure 2(b)).

### 3.2. Growth Factor, Cytokine, and Hyaluronan Concentrations in Platelet-Rich Gel and Platelet-Poor Gel Supernatants

**Figure 3:**
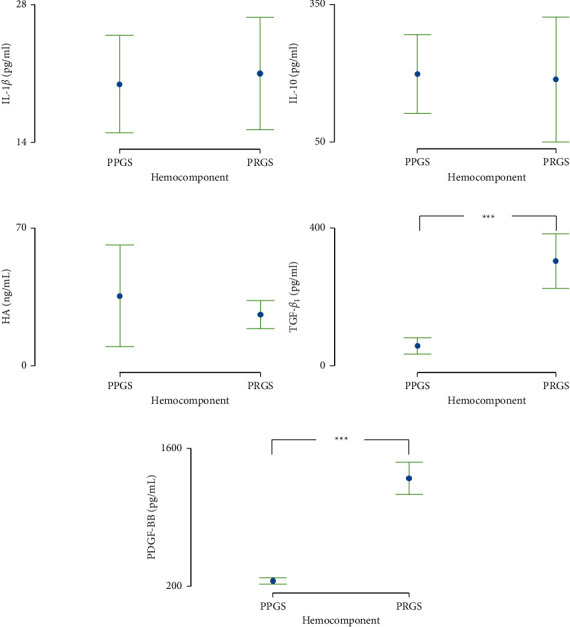
Biomolecule concentrations in both hemocomponents, platelet-poor gel supernatant (PPGS), and platelet-rich gel supernatant (PRGS). (a) Mean (±SD) interleukin 1 beta (IL-1*β*) concentrations in PPGS and PRGS. (b) Mean (±SD) interleukin 10 (IL-10) concentrations in PPGS and PRGS. (c) Mean (±SD) hyaluronan (HA) concentrations in PPGS and PRGS. (d) Mean (±SD) transforming growth factor beta 1 (TGF-*β*_1_) concentrations in PPGS and PRGS. (e) Mean (±SD) platelet-derived growth factor isoform BB (PDGF-BB) concentrations in PPGS and PRGS. Different lower-case letters represent significant (*P* < 0.05) differences between hemocomponents for biomolecules. HA, hyaluronan; IL, interleukin; LPS, lipopolysaccharide; PLT, platelet; PDGF-BB, platelet-derived growth factor isoform BB; PPP/PPGS, platelet-poor plasma/platelet-poor gel supernatant; PRP/PRGS, platelet-rich plasma/platelet-rich gel supernatant; TGF-*β*_1_, transforming growth factor beta 1; WBC, white blood cell.

Basal concentrations of IL-1*β* (Figure 3(a)), IL-10 (Figure 3(b)), and HA (Figure 3(c)) were similar between PPGS and PRGS (Figure 3(a)–3(c)), whereas TGF-*β*_1_ (Figure 3(d)) and PDGF-BB (Figure 3(b)) concentrations were significantly different between both hemocomponents with the higher concentrations for PRGS.

### 3.3. Growth Factor, Cytokine, and Hyaluronan Concentrations in Culture Media at 1 and 48 h

IL-1*β* concentrations were significantly (*P* < 0.001) affected by time, experimental group, and their interaction.

Those experimental groups treated with PPGS at 25% and PRGS at 50% showed a significant increase in these proinflammatory biomolecule concentrations when compared to the same groups at 1 and 48 h. IL-1*β* concentrations were significantly higher in tissue explant groups cultured with PRGS at 50% and PPGS at 25% at 48 h when compared to the remaining experimental groups at any time.

**Figure 4:**
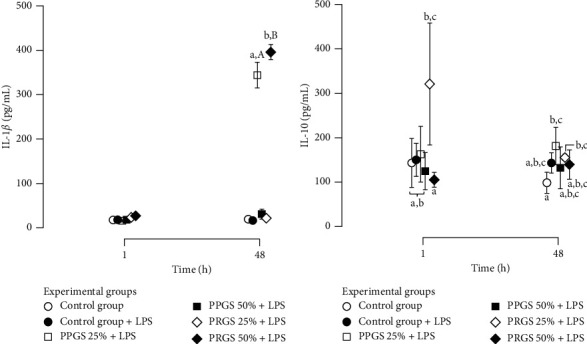
Cytokine concentrations in culture media from experimental groups at 1 and 48 h (a). (a) Mean (±SD) IL-1*β* concentrations (pg/mL) (b) Mean (±SD) IL-10 concentrations (pg/mL). Different lower-case letters represent significant (*P* < 0.01) differences between experimental groups at each independent time. Different capital letters represent significant (*P* < 0.01) differences for the same experimental group at different times. HA, hyaluronan; IL, interleukin; LPS, lipopolysaccharide; PLT, platelet; PDGF-BB, platelet-derived growth factor isoform BB; PPP/PPGS, platelet-poor plasma/platelet-poor gel supernatant; PRP/PRGS, platelet-rich plasma/platelet-rich gel supernatant; TGF-*β*_1_, transforming growth factor beta 1; WBC, white blood cell.

Notably, the concentrations for this catabolic molecule were significantly higher in tissue explants cultured with PRGS at 50% at 48 h than in the culture media of tissue explant cultures with PPGS at 25% at the same time (Figure 4(a)).

IL-10 concentrations were significantly (*P* < 0.001) affected by time, experimental group, and their interaction. All the experimental groups, excluding tissue explants cultured with PRGS at 25%, presented similar concentrations of this anti-inflammatory biomolecule at any time. However, the synovial membranes and CEs cocultured with PRGS at 25% exhibited significantly (*P* < 0.001) higher IL-10 concentrations at 1 h when compared to experimental groups cultured with both PPGS and PRGS at 50% and then significantly (*P* < 0.001) diminished at 48 h (Figure 4(b)).

**Figure 5:**
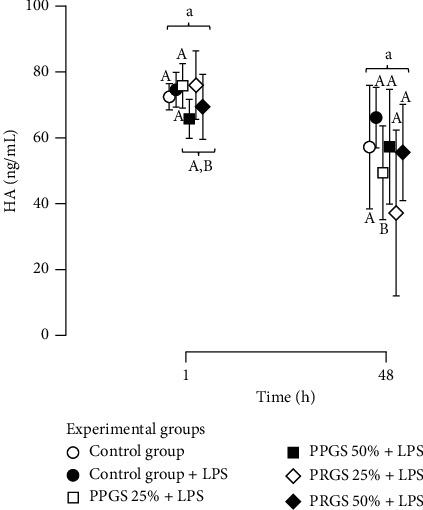
Mean (±SD) HA concentrations (ng/mL) in culture media from experimental groups at 1 and 48 h. Different lower-case letters represent significant (*P* < 0.01) differences between experimental groups at each independent time point. Different capital letters represent significant (*P* < 0.01) differences for the same experimental group at different times. HA, hyaluronan; IL, interleukin; LPS, lipopolysaccharide; PLT, platelet; PDGF-BB, platelet-derived growth factor isoform BB; PPP/PPGS, platelet-poor plasma/platelet-poor gel supernatant; PRP/PRGS, platelet-rich plasma/platelet-rich gel supernatant; TGF-*β*_1_, transforming growth factor beta 1; WBC, white blood cell.

Hyaluronan concentrations in culture media from the experimental groups only were significantly (*P* < 0.001) affected by time and by the interaction of this with the experimental group factor (*P*=0.031). In general, the concentration of this anabolic biomolecule tended to be lower at 48 h when compared to the concentrations obtained in similar experimental groups at 48 h in comparison to both tissue explant control groups and those tissues incubated with PRGS 25% at 1 h. This phenomenon was significantly evident in tissue explants cultured with PPGS at 25% at 1 h (Figure 5).

TGF-*β*_1_ and PDGF-BB concentrations were significantly (*P* < 0.001) affected by the factors experimental group, time, and their interaction. TGF-*β*_1_ concentrations at 1 h were significantly (*P* < 0.001) different between culture media from both control groups, both groups cultured with PPGS at 25% and 50%, and both groups cultured with PRGS at 25% and 50%.

**Figure 6:**
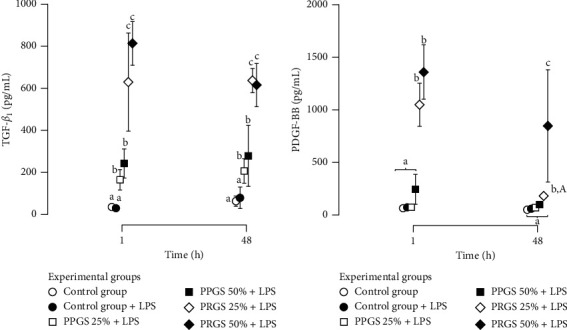
Growth factor concentrations in the culture media of experimental groups at 1 and 48 h (a) Mean (±SD) TGF-*β*_1_ concentrations (pg/mL). (b) Mean (±SD) PDGF-BB concentrations (pg/mL). Different lower-case letters represent significant (*P* < 0.01) differences between experimental groups at each independent time. Different capital letters represent significant (*P* < 0.01) differences for the same experimental group at different times. HA, hyaluronan; IL, interleukin; LPS, lipopolysaccharide; PLT, platelet; PDGF-BB, platelet-derived growth factor isoform BB; PPP/PPGS, platelet-poor plasma/platelet-poor gel supernatant; PRP/PRGS, platelet-rich plasma/platelet-rich gel supernatant; TGF-*β*_1_, transforming growth factor beta 1; WBC, white blood cell.

A similar concentration pattern for this GF was noticed at 48 h; however, although nonsignificant, there was a diminution of the TGF-*β*_1_ concentrations in culture media from tissue explant groups cultured with both PRGS concentrations and a slight increase in the concentrations of this GF in the culture media of tissue explants from both control groups and those cultured with PPGS at both concentrations (Figure 6(a)). On the other hand, at 1 h, PDGF-BB concentrations were significantly higher in the culture media of experimental groups cultured with both PRGS when compared to the remaining experimental groups. However, at 48 h, the concentrations for this GF diminished in the culture media from the tissue explants cultured with both PRGS, particularly at 50%; however, their PDGF-BB concentrations remained significantly higher when compared to the remaining experimental groups (Figure 6(b)).

## 4. Discussion

From a mechanistic perspective, OA has been considered a disease that is unchained by an imbalance between anabolic and catabolic processes that maintain joint homeostasis. Thus, when catabolic factors predominate, the disease becomes more clinically evident [26–28]. There are several biomolecules implicated in maintaining a healthy joint environment, whereas others are overproduced once the OA process is triggered [29]. Bearing this in mind, we selected key biomolecules, such as cytokines (IL-1*β* and IL-10), GFs (TGF-*β*_1_ and PDGF-BB), and the anabolic mediator HA, which are implicated in joint homeostasis and disease to evaluate the effects of two platelet-related hemocomponents (PPRGS and PRGS) to some extent at two concentrations over a short period of time (48 h) in an *in vitro* system that included the coculture of SMEs and CEs challenged with LPS.

IL-1*β* was selected because this proinflammatory and catabolic cytokine has been implicated as one of the main mediators responsible for joint inflammation, cartilage erosion, and chondrocyte aberrant apoptosis [27, 30, 31]. Thus, it has been proposed that the blockade of this catabolic cytokine could be a therapeutic target for controlling or ameliorating OA [31, 32]. However, it is also important to consider that in physiological conditions, this cytokine is important to maintain joint homeostasis through the regulation of matrix metalloproteinases (MMPs) [30]. IL-10 was included in the present study due to its chondroprotective effects in OA tissues by increasing the synthesis of type II collagen and aggrecan and downregulating the expression and secretion of proinflammatory cytokines, such as IL-1*β* and TNF-a. Furthermore, this anti-inflammatory cytokine inhibits chondrocyte apoptosis and the downregulation of MMPs [27].

HA was chosen for the present study because this nonsulfated glycosaminoglycan produced by type B synovial fibroblasts and chondrocytes is of pivotal importance in maintaining joint lubrication and viscoelasticity and improving cartilage compressive stiffness properties [33]. Thus, the production of HA could be considered a potential biomarker for evaluating the effects of the hemocomponents evaluated in the present research. Additionally, we also included the evaluation of TGF-*β*_1_ and PDGF-BB concentrations—which are anabolic and anti-inflammatory biomolecules with positive effects in OA tissues—and why they are contained at supraphysiologic concentrations in platelet-related products [17, 25].

For instance, TGF-*β*_1_ plays several roles in normal cartilage and during the OA process. Under normal conditions, this GF is vital for extracellular matrix (ECM) turnover by increasing the synthesis of type II collagen, aggrecan, and the tissue inhibitors of MMPs (TIMPs). This biomolecule has antagonistic actions on cartilage degradation against catabolic cytokines, such as IL-1*β* and TNF-*α* [28]. On the other hand, PDGF-BB is a mitogenic and anabolic biomolecule for cartilage. It promotes stem cell migration around cartilage defects and increases chondrocyte proliferation and differentiation as well as proteoglycan synthesis. Furthermore, this GF can suppress IL-1*β*-cartilage catabolic effects by downregulating nuclear factor kappa B (NF*κ*B) signaling [31].

The *in vitro* system of cartilage damage (OA) induced by LPS in the present study is a common and widely validated method for elucidating pathogenic mechanisms in the OA process [34] or investigating the effect of several therapeutic substances [35–38], including platelet-related products [39, 40]. In general, LPS can interact with toll-like receptors (TLR) in synovial fibroblasts or chondrocytes (e.g., TLR4 and TLR-IL-1) to—as occurred with proinflammatory cytokines—induce the activation of NF*κ*B transcription, which induces IL-1*β* and TNF-*α* upregulation with subsequent proinflammatory stimuli [20, 34, 41].

The PRP-related product (PRGS) evaluated in the present study is a by-product derived from a platelet concentrate classified as pure PRP, which is characterized by a very low or negligible WBC concentration [42]. Notably, these types of PRP products are more effective in the treatment of arthropathies when compared to PRP preparations rich in leukocytes [15, 16, 43], which are known as leukocyte PRP (L-PRP) [42]. However, an experimental study on dogs found no differences between both PRP products in terms of articular cartilage repair [44]. On the other hand, the PRP evaluated in this study presented PLT counts lower than those reported by previous clinical studies on dogs with OA [15–17]. However, as previously mentioned, the releases of the type platelet concentrate evaluated in the present study presented a significant concentration of mediators with the potential to induce physiological responses in the treated tissues.

According to the reviewed literature, there are no published studies evaluating the effect of PRP products in cartilage, SMEs, isolated chondrocytes, or synovial fibroblasts in dogs. This fact led us to compare the results obtained in the present study with *in vitro* experiments performed on different animal species, such as horses [39, 40] and humans [45]. In line with this, IL-1*β* concentrations at 1 h in the culture media of all evaluated groups remained lower and increased abruptly at 48 h in those tissue explants cultured with PPGS at 25% and PRGS at 50%. The findings of the present study differed from those obtained by Sundman et al. [45], who did not detect IL-1*β* significant concentrations in the culture media from OA human cartilage and synovium cocultured with a P-PRP product at 96 h. The LPS added to culture media from experimental groups in the present research may have resulted in a more robust inflammatory response, which was exacerbated in those tissue explant groups cultured with PPGS at 25% and particularly with PRGS at 50%.

From a mechanistic point of view, the increase in the concentration of this proinflammatory cytokine in culture media of both experimental groups at 48 h could be related with different ways of cell response mediated by the specific concentration of biomolecules contained in both hemocomponents. However, unfortunately, in this in vitro system of canine OA, we measured a limited number of mediators that could be useful to establish if the higher IL-1*β* concentrations were parallelly increased with another pro- or anti-inflammatory biomolecules, such as TNF-*α*, receptor antagonist of IL-1 (IL-1ra), and IL-4.

In line with this, two equine *in vitro* studies [39, 40], which independently evaluated the effect of similar hemocomponents on the synovial membrane and cartilage explants challenged with LPS presented similar TNF-*α* concentrations in culture media at 48 h; however, those tissue explants cultured with a hemocomponent rich in platelets (PRGS) exhibited a better anti-inflammatory profile with increased concentrations on IL-1ra and IL-4 than that produced by the hemocomponent poor in platelets (PPGS). Furthermore, we observed a similar proinflammatory response pattern in ligament explants from horses challenged with LPS and cultured with similar hemocomponents with a biphasic response characterized by a significant increase in IL-1*β* over the first 48 h, followed by a dramatic decline at 96 h [46]. Thus, it is expected that PRGS at 50% can induce an initial robust proinflammatory tissue response that is primarily mediated by higher PDGF-BB concentrations. Unfortunately, the evaluation period of the present study was limited to 48 h.

We observed that the culture media from tissue explants in coculture exhibited very similar IL-10 concentrations at 1 and 48 h, with the exemption of the tissue explant group treated with PRGS at 25%, which presented a higher concentration of this biomolecule over the first hour and then dramatically decreased at 48 h. It is possible that the specific concentration of mediators contained in this hemocomponent at 25% had induced a more robust release of this anti-inflammatory cytokine over the first hour than the rest of the evaluated hemocomponents at different concentrations, perhaps mediated by cell concentration-sensitive receptors of a specific mediator for stimulating IL-10 production. However, it seems to be that despite the production of this cytokine being stimulated by PRGS at 25% over the first hour, there are compensatory mechanisms that induce intake of denaturation of the same over a more prolonged time to maintain it in an optimal concentration, as those observed for anti-inflammatory cytokines, such as IL-4 [39, 40]. This last hypothesis could indicate the reason why the PRP injections in human OA knees did not affect the synovial fluid concentrations of IL-10 over time [47].

On the other hand, we observed that HA was produced by all tissue explant groups in coculture. In general, the production of this mediator may have primarily been stimulated by LPS at 1 h. However, at 48 h, cocultured tissue explants with PPGS at 25% presented a significant lower HA concentration when compared to both control groups and the same hemocomponent at 1 h, which could indicate that this substance at 25% was unable to reverse the catabolic effect of LPS at 48 h. Notably, the HA release pattern from our study was evaluated over a short period of time (only 48 h). This fact could limit the interpretation of our results because the most robust response in HA production in equine joint tissue explants has been observed at 96 h [20, 39]. Thus, it is necessary to conduct new studies evaluating the effects of these types of hemoderivatives in canine joint tissues over a longer period.

We observed that the concentrations of TGF-*β*_1_ in the culture of all cocultured tissue explant groups treated with both hemocomponents at any concentration presented high GF concentrations at 1 h, followed by a significant decrease at 48 h. However, PDGF-BB concentrations, particularly in PRGS at 25%, were dramatically diminished at 48 h. In general, similar concentration patterns for these mediators have been observed in other studies performed by our group in equine cartilage and synovial membrane explants that were cultured independently [39, 40].

The present study has several limitations that must be acknowledged to improve the understanding of readers. First, this is an *in vitro* study involving a biological system that includes some pieces of the bigger puzzle representing a patient or joint affected by OA. Notably, it provides useful information to inform more rational future studies of OA animal models and OA patients. Generally, the results of the present study were unexpected because all evaluated hemocomponents produced limited anti-inflammatory effects and some of them (PPGS at 25% and PPRGS at 50%) were even markedly proinflammatory. The reason for these controversial findings could be related to the short duration (only 48 h) of the study, which could have limited the biological process implicated in the production of the evaluated biomolecules. Thus, future studies should be performed over longer periods.

## 5. Conclusions

At both concentrations, both PPGS and PRGS showed limited biological effects on cartilage and SMEs in coculture with LPS, while even PPGS at 25% and PRGS at 50% exhibited proinflammatory effects on these tissues at 48 h. Notably, further studies should be performed to evaluate the effects of both hemoderivatives over longer study periods.

## Data Availability

The data used in this research are available upon request to the corresponding author.

## References

[1] Anderson K. L., O’Neill D. G., Brodbelt D. C. (2018). Prevalence, duration and risk factors for appendicular osteoarthritis in a UK dog population under primary veterinary care. *Scientific Reports*.

[2] Mehana E.-S. E., Khafaga A. F., El-Blehi S. S. (2019). The role of matrix metalloproteinases in osteoarthritis pathogenesis: an updated review. *Life Sciences*.

[3] Cimino Brown D. (2017). What can we learn from osteoarthritis pain in companion animals?. *Clinical & Experimental Rheumatology*.

[4] Johnston S. A. (1997). Osteoarthritis. *Veterinary Clinics of North America: Small Animal Practice*.

[5] Neil D. G. O., Church D. B., McGreevy P. D., Thomson P. C., Brodbelt D. C. (2014). Prevalence of disorders recorded in dogs attending primary-care veterinary practices in England. *PLoS One*.

[6] Clements D. N., Carter S. D., Innes J. F., Ollier W. E. R. (2006). Genetic basis of secondary osteoarthritis in dogs with joint dysplasia. *American Journal of Veterinary Research*.

[7] Bhathal A., Spryszak M., Louizos C., Frankel G. (2017). Glucosamine and chondroitin use in canines for osteoarthritis: a review. *Open Veterinary Journal*.

[8] Black L. L., Gaynor J., Adams C. (2008). Effect of intraarticular injection of autologous adipose-derived mesenchymal stem and regenerative cells on clinical signs of chronic osteoarthritis of the elbow joint in dogs. *Veterinary Therapeutics: Research in Applied Veterinary Medicine*.

[9] Kim S. E., Pozzi A., Yeh J.-c. (2019). Intra-articular umbilical cord derived mesenchymal stem cell therapy for chronic elbow osteoarthritis in dogs: a double-blinded, placebo-controlled clinical trial. *Frontiers in Veterinary Science*.

[10] Maki C. B., Beck A., Wallis C.-B. C. C. (2020). Intra-articular administration of allogeneic adipose derived MSCs reduces pain and lameness in dogs with hip osteoarthritis: a double blinded, randomized, placebo controlled pilot study. *Frontiers in Veterinary Science*.

[11] Pavarotti G. S., Hivernaud V., Brincin M. (2020). Evaluation of a single intra-articular injection of autologous adipose tissue for the treatment of osteoarthritis: a prospective clinical study in dogs. *Veterinary and Comparative Orthopaedics and Traumatology: V.C.O.T*.

[12] Srzentić Dražilov S., Mrkovački J., Spasovski V., Fazlagić A., Pavlović S., Nikčević G. (2018). The use of canine mesenchymal stem cells for the autologous treatment of osteoarthritis. *Acta Veterinaria Hungarica*.

[13] Catarino J., Carvalho P., Santos S., Martins Â., Requicha J. (2020). Treatment of canine osteoarthritis with allogeneic platelet-rich plasma: review of five cases. *Open Veterinary Journal*.

[14] Cuervo B., Rubio M., Chicharro D. (2020). Objective comparison between platelet rich plasma alone and in combination with physical therapy in dogs with osteoarthritis caused by hip dysplasia. *Animals: An Open Access Journal from MDPI*.

[15] Venator K., Frye C. W., Gamble L.-J., Wakshlag J. J. (2020). Assessment of a single intra-articular stifle injection of pure platelet rich plasma on symmetry indices in dogs with unilateral or bilateral stifle osteoarthritis from long-term medically managed cranial cruciate ligament disease. *Veterinary Medicine: Research and Reports*.

[16] Vilar J. M., Manera M. E., Santana A. (2018). Effect of leukocyte-reduced platelet-rich plasma on osteoarthritis caused by cranial cruciate ligament rupture: a canine gait analysis model. *PLoS One*.

[17] Silva R. F., Carmona J. U., Rezende C. M. (2013). Intra-articular injections of autologous platelet concentrates in dogs with surgical reparation of cranial cruciate ligament rupture: a pilot study. *Veterinary and Comparative Orthopaedics and Traumatology: V.C.O.T*.

[18] AVMA Panel on Euthanasia (2001). 2000 report of the AVMA panel on euthanasia. *Journal of the American Veterinary Medical Association*.

[19] Congress C., Congress C. (2002). Law by which the possession and registration of potentially dangerous dogs is regulated. *Official Bulletin of the State*.

[20] Carmona J. U., Ríos D. L., López C., Álvarez M. E., Pérez J. E., Bohórquez M. E. (2016). In vitro effects of platelet-rich gel supernatants on histology and chondrocyte apoptosis scores, hyaluronan release and gene expression of equine cartilage explants challenged with lipopolysaccharide. *BMC Veterinary Research*.

[21] Carmona J. U., Ríos D. L., López C., Álvarez M. E., Pérez J. E. (2017). Proinflammatory and anabolic gene expression effects of platelet-rich gel supernatants on equine synovial membrane explants challenged with lipopolysaccharide. *Veterinary Medicine International*.

[22] Moreira M. L., Dorneles E. M. S., Soares R. P. (2015). Cross-reactivity of commercially available anti-human monoclonal antibodies with canine cytokines: establishment of a reliable panel to detect the functional profile of peripheral blood lymphocytes by intracytoplasmic staining. *Acta Veterinaria Scandinavica*.

[23] Manning A. M., Auchampach J. A., Drong R. F., Slightom J. L. (1995). Cloning of a canine cDNA homologous to the human transforming growth factor-*β*1-encoding gene. *Gene*.

[24] Franklin S. P., Birdwhistell K. E., Strelchik A., Garner B. C., Brainard B. M. (2017). Influence of cellular composition and exogenous activation on growth factor and cytokine concentrations in canine platelet-rich plasmas. *Frontiers in Veterinary Science*.

[25] Silva R. F., Carmona J. U., Rezende C. M. (2012). Comparison of the effect of calcium gluconate and batroxobin on the release of transforming growth factor beta 1 in canine platelet concentrates. *BMC Veterinary Research*.

[26] Gossan N., Boot-Handford R., Meng Q.-J. (2015). Ageing and osteoarthritis: a circadian rhythm connection. *Biogerontology*.

[27] Wojdasiewicz P., Poniatowski Ł. A., Szukiewicz D. (2014). The role of inflammatory and anti-inflammatory cytokines in the pathogenesis of osteoarthritis. *Mediators of Inflammation*.

[28] Finnson K. W., Chi Y., Bou-Gharios G., Leask A., Philip A. (2012). TGF-beta signaling in cartilage homeostasis and osteoarthritis. *Frontiers in Bioscience*.

[29] Martel-Pelletier J., Barr A. J., Cicuttini F. M. (2016). Osteoarthritis. *Nature Reviews Disease Primers*.

[30] Wang T., He C. (2018). Pro-inflammatory cytokines: the link between obesity and osteoarthritis. *Cytokine & Growth Factor Reviews*.

[31] Montaseri A., Busch F., Mobasheri A. (2011). IGF-1 and PDGF-bb suppress IL-1*β*-induced cartilage degradation through down-regulation of NF-*κ*B signaling: involvement of src/PI-3K/AKT pathway. *PLoS One*.

[32] Conaghan P. G., Cook A. D., Hamilton J. A., Tak P. P. (2019). Therapeutic options for targeting inflammatory osteoarthritis pain. *Nature Reviews Rheumatology*.

[33] Kuroki K., Cook J. L., Kreeger J. M. (2002). Mechanisms of action and potential uses of hyaluronan in dogs with osteoarthritis. *Journal of the American Veterinary Medical Association*.

[34] Barreto G., Senturk B., Colombo L. (2020). Lumican is upregulated in osteoarthritis and contributes to TLR4-induced pro-inflammatory activation of cartilage degradation and macrophage polarization. *Osteoarthritis and Cartilage*.

[35] Li X., Zhang Z., Liang W. (2020). Tougu Xiaotong capsules may inhibit p38 MAPK pathway-mediated inflammation: in vivo and in vitro verification. *Journal of Ethnopharmacology*.

[36] Li Y., Li K., Hu Y., Xu B., Zhao J. (2015). Piperine mediates LPS induced inflammatory and catabolic effects in rat intervertebral disc. *International Journal of Clinical and Experimental Pathology*.

[37] Hartog A., Hougee S., Faber J. (2008). The multicomponent phytopharmaceutical SKI306X inhibits in vitro cartilage degradation and the production of inflammatory mediators. *Phytomedicine*.

[38] Hu H., Li Y., Xin Z., Zhang X. (2018). Ginkgolide B exerts anti-inflammatory and chondroprotective activity in LPS-induced chondrocytes. *Advances in Clinical and Experimental Medicine*.

[39] Ríos D. L., López C., Álvarez M. E., Samudio I. J., Carmona J. U. (2015). Effects over time of two platelet gel supernatants on growth factor, cytokine and hyaluronan concentrations in normal synovial membrane explants challenged with lipopolysaccharide. *BMC Musculoskeletal Disorders*.

[40] Ríos D. L., López C., Carmona J. U. (2015). Evaluation of the anti-inflammatory effects of two platelet-rich gel supernatants in an in vitro system of cartilage inflammation. *Cytokine*.

[41] Zeddou M. (2019). Osteoarthritis is a low-grade inflammatory disease: obesity’s involvement and herbal treatment. *Evidence-based Complementary and Alternative Medicine: eCAM*.

[42] Dohan Ehrenfest D. M., Andia I., Zumstein M. A., Zhang C.-Q., Pinto N. R., Bielecki T. (2014). Classification of platelet concentrates (Platelet-Rich Plasma-PRP, Platelet-Rich Fibrin-PRF) for topical and infiltrative use in orthopedic and sports medicine: current consensus, clinical implications and perspectives. *Muscles Ligaments Tendons Journal*.

[43] Araya N., Miyatake K., Tsuji K. (2020). Intra-articular injection of pure platelet-rich plasma is the most effective treatment for joint pain by modulating synovial inflammation and calcitonin gene-related peptide expression in a rat arthritis model. *The American Journal of Sports Medicine*.

[44] Kazemi D., Fakhrjou A. (2015). Leukocyte and platelet rich plasma (L-PRP) versus leukocyte and platelet rich fibrin (L-PRF) for articular cartilage repair of the knee: a comparative evaluation in an animal model. *Iranian Red Crescent Medical Journal*.

[45] Sundman E. A., Cole B. J., Karas V. (2014). The anti-inflammatory and matrix restorative mechanisms of platelet-rich plasma in osteoarthritis. *The American Journal of Sports Medicine*.

[46] Castillo-Franz C., López C., Álvarez M. E., Giraldo C. E., Carmona J. U. (2019). *Muscles Ligaments Tendons Journal*.

[47] Mariani E., Canella V., Cattini L. (2016). Leukocyte-rich platelet-rich plasma injections do not up-modulate intra-articular pro-inflammatory cytokines in the osteoarthritic knee. *PLoS One*.

